# Artificial Intelligence Literacy Levels of Perioperative Nurses: The Case of Türkiye

**DOI:** 10.1111/nhs.70059

**Published:** 2025-02-13

**Authors:** Hilal Kahraman, Seda Akutay, Hatice Yüceler Kaçmaz, Sultan Taşci

**Affiliations:** ^1^ Department of Nursing, Faculty of Health Sciences Erciyes University Türkiye

**Keywords:** artificial intelligence, artificial intelligence literacy, healthcare, perioperative care, perioperative nurses

## Abstract

Artificial intelligence (AI) experience among nurses in perioperative settings is crucial for effective healthcare delivery. This study aimed to assess AI literacy levels and associated characteristics among perioperative nurses in Türkiye. This cross‐sectional study was conducted between March 15 and April 15, 2024, and included 505 perioperative nurses. Data were collected online using the “Nurse Information Form” and “AI Literacy Scale.” Snowball sampling technique was used to enlist individuals. The mean literacy score was 44.35 ± 5.88, which means moderate proficiency. Key elements associated with high literacy levels included being male, familiarity with and use of AI applications, youth, seeing AI as a tool to alleviate workload, and using information technology tools. The results show that although perioperative nurses have a moderate level of AI literacy, their use of AI tools is minimal. The findings underline the need for professional development in AI integration and the inclusion of relevant materials in nursing education programs.

**Summary**

- The use of artificial intelligence (AI) is increasing in healthcare, just as it is in many other fields. As AI becomes more prevalent, the demand for knowledge, skills, and practical applications also grows.
- Given the number and roles of personnel within the healthcare system, it is anticipated that nurses will be the group most affected by this trend.
- With the expanding use of AI, it is essential to identify the knowledge, attitudes, and needs of healthcare personnel to better understand how to integrate and utilize these technologies effectively.

## 1 Introduction

Artificial intelligence (AI) is a broad conceptual definition based on teaching computers to imitate human functions such as reasoning, communication, and decision‐making (Clancy 2020; Von Gerich et al. 2022; McGrow 2019; Polatgil and Güler 2023; Robert 2019). AI, whose foundations date back to 1956, has gained significant momentum as of 2011 with increased computer capabilities and the availability of extensive data sets to train AI systems (AI‐HLEG 2019; Robert 2019). The rapid development of AI in recent years has brought transformation in healthcare services. For example, robots in patient care can monitor patient behavior, detect situational abnormalities, and notify nurses. Similarly, developed robots can be used in various care functions such as mobilization support, drug dosage adjustment, and administration (Akgerman et al. 2022; Macfie 2014). Considering the potential effects of these technological developments on patient care, the importance of nurses having a certain level of knowledge and skills regarding AI also emerges (Alderden 2023; Aygin and Gül 2021; McGrow 2019).

The first data on AI in nursing was found in the Medline database in 1985 (Ryan 1985). As in many fields, the use of AI in nursing has gained momentum in the last 10 years (Aygin and Gül 2021; Von Gerich et al. 2022). Today, the data ecosystem suitable for use in the field of nursing is very rich and offers many possibilities for AI, promising to reduce costs and increase the efficiency of health services (Alderden 2023; Von Gerich et al. 2022).

AI is progressing rapidly and will progress in the surgical field, where nurses play an active role. Including AI in the surgical decision‐making process will contribute to the transformation in care by changing the decision of surgery, informed consent/consent process, reducing modifiable risk factors, managing postoperative complications, care, and joint decisions on resource use. It will be used more widely with the enrichment of data in both preoperative and postoperative settings (Chadebecq et al. 2023; Loftus et al. 2020; Ölçer and Yılmaz 2021). Robots equipped with AI software in surgery help reduce the physical workload of nurses. Robots equipped with AI software in the surgical field help reduce nurses' physical workload (Akgerman et al. 2022). For example, Locsin and Ito (2018), stated that the Da Vinci surgeon‐controlled robot contributed to reducing the responsibilities of operating room nurses and increasing the efficiency of surgery by eliminating the anatomical limitations of human hands during surgery (Locsin and Ito 2018). Another example of nursing care in AI is the development of a polar bear‐like care robot named Ro‐bear. Ro‐bear robots can put patients in bed or a wheelchair, lift them, and support mobilization (Akgerman et al. 2022; Macfie 2014). The IV Robot RIVA can prepare and administer the correct dose of intravenous medication (Kandemir et al. 2023).

The high workload of surgical nurses (Karacabay et al. 2020; Nawrat 2023; Ugurlu et al. 2015) suggests that the need for AI support will increase. However, it is stated in the literature that the participation and use of AI by nurses who take an active role in this field are insufficient (Abdullah and Fakieh 2020; Fritz and Dermody 2019; O'Connor et al. 2023). The increasing tendency toward using AI brings about the need for change and development in the knowledge and skill levels of nurses who are surgical team members in this field. Increasing the level of AI knowledge in nurses will contribute to the development and increased use of AI applications in care (Aygin and Gül 2021; O'Connor et al. 2023). However, it is necessary to reveal nurses' level of AI knowledge and related factors. In this context, no study was found in which nurses' AI literacy level was evaluated when the literature was examined. This study aims to determine the level of AI literacy and related factors of perioperative nurses. The study's results are thought to contribute to planning nursing education, research, and practice in Türkiye.

## 2 Materials and Methods

### 2.1 Study Design

This study was conducted using a descriptive design. This study followed the relevant EQUATOR guidelines and named the reporting method. The Strengthening the Reporting of Observational Studies (STROBE) checklist for cross‐sectional studies was used to guide reporting.

The cross‐sectional design was chosen as it aligned well with the study's objective of assessing perioperative nurses' use of AI and their literacy levels at a specific point in time. This design allows data to be collected from a large sample over a limited period of time, enabling the identification of participants' current knowledge, practices and perceptions of AI. The design captures data across the target audience simultaneously, enabling effective identification of trends, relationships, and areas requiring intervention. In addition, adherence to the STROBE checklist ensures methodological transparency and rigor, further supporting the reliability and validity of the findings. This approach effectively meets the objectives of the study by providing a comprehensive understanding of the current state of AI‐related competences among perioperative nurses.

### 2.2 Population and Sample

**FIGURE 1:**
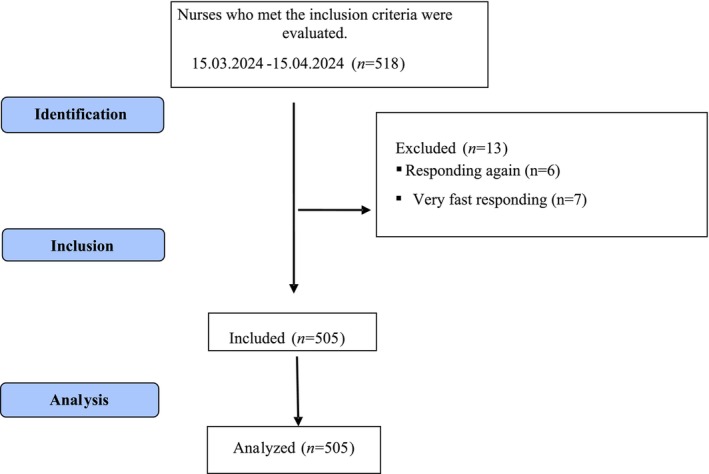
Flow chart.

The study population comprised perioperative nurses working in Türkiye between March 15, 2024, and April 15, 2024. Perioperative nurses work in operating theater, surgical clinic, surgical intensive care unit, surgical outpatient clinic and emergency departments. In the study, the formula [*z*2 *p* (100 − *p*)/*e*2] was used to calculate the sample whose population was unknown. According to the formula, the minimum sample size was determined as 385 with a *z* score of 1.96 (*z*) at a 95% confidence interval, a reliable sample size of 50% (*p*) (Liu et al. 2021), and a 5% margin of error (*e*). The snowball sampling method was used to reach the sample size. The researchers reached the nurses via mobile phone, e‐mail, or social media. In addition, the nurses participating in the study were asked to deliver the questionnaire to the nurses around them. The study reached 518 nurses. Six of the nurses gave repeated answers and seven of them answered very quickly, so these data were not included in the study. The study was completed with 505 questionnaires (Figure 1).

The inclusion and exclusion criteria of the research are as follows:

*Criteria for inclusion*—

− Working as a nurse,
  − Working in a surgical clinic/operating room for at least 6 months,
  − Volunteering to participate in the research.

*Criteria for exclusion*

− Not during leave (annual leave/maternity leave/unpaid leave).

### 2.3 Data Collection

The study data were collected between March 15, 2024, and April 15, 2024, with an online survey application.

The data was collected using the “AI Literacy Scale” (Polatgil and Güler 2023) and the “Personal Information Form” created by the authors in line with the literature (Von Gerich et al. 2022; Kandemir et al. 2023; Yang et al. 2022).

### 2.4 Data Collection Tools

#### 2.4.1 Nurse Information Form

In the personal information form, there are eight questions in total regarding age, gender, level of education, cynicism, use of AI, the status of receiving training on AI, the idea of AI to reduce the workload of nurses, and the ability to use technology tools (Von Gerich et al. 2022; Kandemir et al. 2023; Liu et al. 2021; Polatgil and Güler 2023).

#### 2.4.2 AI Literacy Scale

In the study, the scale developed by Wang, Rau, and Yuan (2023) and the Turkish validity and reliability study conducted by Polatgil and Güler (2023) consists of a total of 12 items. The scale has four subdimensions: Awareness (three items), Use (three items), Evaluation (three items), Ethics (three items). Awareness: Perioperative nurses' level of awareness of AI technologies helps them understand the role of these technologies in patient care. This subdimension determines the extent to which nurses have knowledge before adopting new technologies and how they are ready to use these technologies. Utilization: This subdimension determines the extent to which nurses use AI tools in practice and the effectiveness of their use. In perioperative nursing practice, ethical responsibilities such as confidentiality, autonomy, and correct data use regarding patient information are very important. The ethical subdimension evaluates the nurses' handling of such ethical issues that they may encounter with AI applications. Evaluation: this subdimension evaluates the effectiveness and reliability of AI systems and the ability of perioperative nurses to make correct decisions. The scale is a 7‐point Likert‐type scale with response options such as “strongly agree, agree, somewhat agree, undecided, somewhat disagree, disagree, strongly disagree.” There are three reverse‐scored items in the scale. A minimum score of 12 and a maximum score of 84 can be obtained from the scale. As the score increases, it means that the person's AI literacy level increases. In the Turkish validity and reliability study of the scale, the total scale Cronbach's *α* value was 0.939; in this study, the total scale Cronbach's *α* value was 0.821.

### 2.5 Data Analysis

The SPSS 24 was used for the statistical analysis of the data. Frequency and percentage for categorical data and mean and standard deviation for continuous numerical data were used as descriptive statistics. The suitability of the data for normal distribution was tested with kurtosis and skewness. Due to the normal distribution of the data, an independent two‐sample *t* test, dependent sample *t* test, and Pearson's correlation analysis were used. A value of *p* < 0.05 was considered statistically significant in the data evaluation.

## 3 Results

The study involved 505 perioperative nurses. The mean age of the nurses participating in the study was 31.55 ± 7.70 years. Of the nurses participating in the study, 70.0% were female, 76.8% had a bachelor's degree, and 37.0% worked in the surgical clinical unit. 1.6% of the nurses stated that received training on AI. The nurses who received the training stated that they received it from the institution where they worked and/or during postgraduate education. It was determined that Chatgbt was the most well‐known AI application. 16.4% of perioperative nurses said they used AI applications, and 78.0% thought AI would reduce nurses' workload. 43.0% of the nurses stated that their use of information technology tools was good (Table 1).

**TABLE 1:** Sociodemographic and artificial intelligence usage characteristics of nurses (*n* = 505).

Characteristics	*n*	%
Sex		
Female	399	79.0
Male	106	21.0
Education level		
High school	41	8.2
Undergraduate	388	76.8
Postgraduate	76	15.0
Unit of work		
Operating room	67	13.3
Surgical clinic	187	37.0
Surgical intensive care	121	24.0
Surgical outpatient clinic	43	8.5
Emergency service	87	17.2
Receiving training on AI		
Yes	8	1.6
No	497	98.4
Hearing AI applications		
Yes	162	32.1
No	343	67.9
Previously knew AI applications^a^		
Chatgbt	146	28.9
Murf	12	2.4
İmage‐Bind	10	2.0
Bing‐Chat	20	4.0
DALL‐E	17	3.4
Synthesia	8	1.6
Pictory	19	3.8
SIMA	1	0.2
Sora	1	0.2
Use of previously used AI applications		
Yes	83	16.4
No	422	83.6
Previously used AI applications^a^		
Chatgbt	77	15.2
Murf	3	0.6
Image‐Bind	1	0.2
Bing‐Chat	2	0.4
DALL‐E	7	1.4
Synthesia	2	0.4
Pictory	6	1.2
Thinking that AI will reduce the workload of the nurse		
Yes	394	78.0
No	111	22.0
Use of information technology tools		
Very good (advanced)	93	18.4
Good	217	43.0
Medium	172	34.1
Low	17	3.4
Very low	6	1.2
Age		
*X−* ± *SS* (Min–max)	31.55 ± 7.70	(20–56)

Abbreviation: AI, artificial intelligence.

^a^ More than one option is selected.

When the AI Literacy Scale scores of the nurses were evaluated, it was found that the mean score of the awareness subdimension was 10.22 ± 1.94, the mean score of the usage subdimension was 10.71 ± 2.04, the mean score of the evaluation subdimension was 11.47 ± 1.82, the mean score of the ethical subdimension was 11.94 ± 1.88 and the mean score of the total scale was 44.35 ± 5.88 (Table 2).

**TABLE 2:** Artificial Intelligence Literacy Scale and subscale score of nurses.

Subscales	*X−± SS*	95% confidence interval	(Min–max)
Awareness	10.22 ± 1.94	10.05–10.29	4.00–15.00
Use	10.71 ± 2.04	10.53–10.88	4.00–15.00
Evaluation	11.47 ± 1.82	11.31–11.63	5.00–15.00
Ethics	11.94 ± 1.88	11.77–12.10	6.00–15.00
Total score	44.35 ± 5.88	43.83–44.86	28.00–60.00

When the relationship between the descriptive characteristics of the nurses and the scale and subdimension scores of AI was analyzed, it was found that the mean scores of awareness, use, evaluation, and total scale were significantly higher in males than females (*p* < 0.05). It was determined that the awareness, usage, evaluation, and total scale mean scores of those who heard about AI applications were significantly higher (*p* < 0.05). It was found that the mean scores of awareness, use, evaluation, ethical subdimension, and total scale scores of nurses who stated that they used AI applications were significantly higher than those who did not use them (*p* < 0.05). The awareness, usage, evaluation, ethics, and total scale scores of nurses who think that AI will reduce the workload of nurses are higher than those who do not. This difference between the groups is statistically significant (*p* < 0.05). The awareness subdimension scores of the nurses who were very good at using information technology tools were higher than those who were good, good than those who were medium, and medium than those who were poor. This difference between the groups was statistically significant (*p* < 0.05). The use subdimension scores of the nurses who were very good at using information technology tools were statistically significantly higher than those who were good, medium, poor, and very poor. The mean scores of the evaluation subdimension nurses who were very good at using information technology tools were significantly higher than the others (*p* < 0.05). The ethical subdimension mean scores of the nurses with good status of using information technology tools are significantly higher than those with very good status and significantly lower than those with poor status. It was determined that the mean total scale score of the nurses who were very good at using information technology tools was statistically significantly higher than the others (*p* < 0.05). As the age of the nurses increased, the mean scores of awareness, use, evaluation, and total scale scores decreased significantly (*p* < 0.05) (Table 3).

**TABLE 3:** Comparison between characteristics of nurses and Artificial Intelligence Literacy Scale scores.

Characterictics	AI Literacy Scale				
	Awareness	Use	Evaluation	Ethic	Total scale
Sex					
Female	10.04 ± 1.89	10.59 ± 1.99	11.36 ± 1.78	11.91 ± 1.77	43.91 ± 5.64
Male	10.91 ± 1.98	11.15 ± 2.18	11.87 ± 1.93	12.03 ± 2.42	45.98 ± 6.48
*p* ^a^	< **0.001**	**0.013**	**0.010**	0.558	**0.001**
Education level					
High school	10.24 ± 2.04	10.51 ± 2.29	11.34 ± 1.94	11.48 ± 1.98	43.58 ± 6.13
Undergraduate	10.12 ± 1.91	10.64 ± 1.99	11.49 ± 1.80	11.93 ± 1.89	44.19 ± 5.75
Postgraduate	10.71 ± 2.00	11.15 ± 2.11	11.44 ± 1.88	12.22 ± 1.71	45.53 ± 6.29
*p* ^b^	0.056	0.109	0.874	0.129	0.131
Unit of work					
Operating room	9.91 ± 1.79	10.41 ± 2.16	11.14 ± 1.70	11.88 ± 1.96	43.35 ± 5.55
Surgical clinic	10.17 ± 2.03	10.77 ± 2.08	11.59 ± 1.93	12.04 ± 1.79	44.58 ± 6.12
Surgical intensive care	10.39 ± 1.94	10.56 ± 1.71	11.32 ± 1.53	11.74 ± 1.81	44.02 ± 5.17
Surgical outpatient clinic	10.23 ± 1.90	10.90 ± 1.91	11.34 ± 2.24	11.58 ± 2.16	44.06 ± 6.13
Emergency service	10.32 ± 1.90	10.91 ± 2.33	11.73 ± 1.80	12.21 ± 1.91	45.19 ± 6.34
*p* ^b^	0.554	0.484	0.221	0.243	0.346
Receiving training on AI					
Yes	10.87 ± 2.85	11.50 ± 2.61	12.12 ± 2.85	11.62 ± 2.38	46.12 ± 8.30
No	10.21 ± 1.92	10.69 ± 2.03	11.46 ± 1.81	11.94 ± 1.87	44.32 ± 5.84
*p* ^a^	0.534	0.417	0.533	0.715	0.560
Knowing AI applications					
Yes	10.85 ± 2.04	11.03 ± 2.16	11.85 ± 1.84	12.16 ± 1.87	45.90 ± 6.00
No	9.92 ± 1.82	10.55 ± 1.97	11.29 ± 1.79	11.83 ± 1.87	43.61 ± 5.68
*p* ^a^	< **0.001**	**0.016**	**0.001**	0.074	< **0.001**
Use of previously used AI applications					
Yes	11.34 ± 2.17	11.45 ± 2.07	12.09 ± 1.86	12.31 ± 1.89	47.21 ± 6.02
No	10.00 ± 1.81	10.56 ± 2.01	11.35 ± 1.79	11.86 ± 1.87	43.78 ± 5.69
*p* ^a^	< **0.0001**	**< 0.0001**	**0.001**	**0.049**	**< 0.001**
Thinking that AI will reduce the workload of the nurse					
Yes	10.37 ± 1.97	10.95 ± 1.96	11.69 ± 1.76	12.11 ± 1.81	45.13 ± 5.72
No	9.68 ± 1.73	9.83 ± 2.10	10.70 ± 1.84	11.33 ± 1.99	41.55 ± 5.58
*p* ^a^	**0.001**	< **0.001**	**< 0.001**	< **0.001**	< **0.001**
Use of information technology tools					
Very good (advanced)	11.43 ± 2.13^a^	11.95 ± 2.08	12.66 ± 1.92	12.69 ± 2.10	48.75 ± 6.31
Good	10.43 ± 1.58^a^	11.04 ± 1.68	11.50 ± 1.54	11.98 ± 1.78	44.96 ± 4.3
Medium	9.51 ± 1.76^a^	9.90 ± 1.78	11.00 ± 1.64	11.59 ± 1.72	42.01 ± 4.77
Low	8.17 ± 1.42	8.47 ± 1.77	9.58 ± 2.23	10.64 ± 1.83	36.88 ± 3.98
Very low	9.83 ± 3.76^a^	9.00 ± 4.85	10.83 ± 3.31	12.33 ± 1.96	42.00 ± 12.11
*p* ^b^	< **0.001**	< **0.001**	< **0.001**	< **0.001**	< **0.001**
Age (*X* ± SD/median)	*r* = −0.179	*r* = −0.140	*r* = −0.176	*r* = −0.061	*r* = 0.182
*p* ^c^	< **0.001**	**0.002**	< **0.001**	0.172	< **0.001**

*Note:* The bold values represent statistically significant difference at *p* < 0.05.

Abbreviations: AI, artificial intelligence.

^a^ Independent samples *t* test.

^b^ One‐way variance analysis.

^c^ Pearson's correlation test.

When the correlation of the subdimension and total score averages of the nurses' AI Literacy Scale was examined, it was found that there was a moderate positive correlation between the awareness subdimension mean score and the use subdimension mean score (*r* = 0.478, *p* < 0.001) and the evaluation subdimension mean score (*r* = 0.425, *p* < 0.001), a weak positive correlation with the ethical subdimension mean score (*r* = 0.270, *p* < 0.001), and a strong positive correlation with the total scale mean score. There was a moderate positive correlation between the mean score of the utilization subdimension and the mean score of the evaluation subdimension (*r* = 0.622, *p* < 0.001) and a strong positive correlation with the mean score of the total scale. There was a statistically significant correlation between the mean score of the evaluation subdimension and the mean score of the ethical subdimension (*r* = 0.490, *p* < 0.001) at a moderate positive level and between the mean score of the total scale (*r* = 0.825, *p* < 0.001) at a substantial level. There was a statistically significant positive and moderate correlation between the mean score of the ethics subdimension and the mean score of the total scale (*r* = 0.693, *p* < 0.001) (Table 4).

**TABLE 4:** Correlation between nurses' Artificial Intelligence Literacy Scale subscale scores.

Subscales	Awareness	Use	Evaluation	Ethic
Awareness	—			
Use	*r* = 0.478^a^ *p* **< 0.001**			
Evaluation	*r* = 0.425^a^ *p* < **0.001**	*r* = 0.622^a^ *p* < **0.001**		
Ethic	*r* = 0.270^a^ *p* < **0.001**	*r* = 0.378^a^ *p* < **0.0001**	*r* = 0.490^a^ *p* < **0.001**	
Total scale	*r* = 0.715^a^ *p* < **0.001**	*r* = 0.820^a^ *p* < **0.001**	*r* = 0.825^a^ *p* < **0.001**	*r* = 0.693^a^ *p* < **0.001**

*Note:* The bold values represent statistically significant difference at *p* < 0.05.

^a^ Pearson's correlation test.

## 4 Discussion

As in every field, the need for health services is increasing daily. This situation brings along the need for knowledge, skills, and applications related to AI. Considering the number and function of personnel in the health system, the group most affected by this situation will be nurses. In this study, which was conducted to determine the AI literacy levels of nurses and related factors, it was determined that nurses' AI literacy levels were at a medium level, and the use of AI was relatively low.

In the study, male nurses had higher AI literacy levels than female nurses except for the ethical subdimension. Although there are no studies evaluating the relationship between AI and gender in studies conducted with nurses, there are results similar to the data of this study in studies conducted with different groups. For example, a study conducted with high school students reported that female students had lower AI autonomy, competence, and knowledge levels than male students and needed more support (Xia et al. 2023). Similarly, there are studies in the literature reporting that the level of knowledge and use of AI is higher in men than in women (Al Hadithy et al. 2023; Badaloni and Rodà 2022; Buslón et al. 2023; Chen et al. 2021). However, a study with undergraduate students found that female students' sensitivity levels were higher in the subdimensions of justice, privacy, and nonharm regarding AI (Jang et al. 2022). Considering that the majority of the nurse population consists of women, it is clear that the group needs support. It can be said that policies are needed to ensure social equality in AI.

Our study data show that AI literacy decreases with increasing age. Data in the literature support this finding and show age‐related prejudices (Chu et al. 2023; Dev and Phillips 2019; Díaz et al. 2017). This situation thinks that the use of technological devices is increasing daily, and as the age range decreases, these devices become almost a necessity. The use of technology in education, especially with distance education during the COVID‐19 pandemic, did not allow young people to continue their education without using technological tools. For this reason, the machine learning of the young generation has necessarily developed the use of technological tools. AI and machine learning are complementary technologies and are transforming the way modern devices work. AI is a field of technology that performs functions such as decision making, problem solving and learning by imitating human intelligence. Machine learning, as a subbranch of AI, enables devices to learn from data to improve their performance in a given task. This relationship allows technological devices to become more intelligent, user‐friendly and customisable, making daily life easier (Gupta et al. 2021; Theodosiou and Read 2023). In light of these data, it is thought that exposure to technological tools and AI with age will affect literacy level. For this reason, it should be considered that including applied training in clinics will profoundly contribute to the level of AI literacy.

The rate of nurses hearing about AI applications is 32.1%, and the rate of use is 16.4%. These results are pretty low considering the rapid introduction of AI into daily life. Similar to the results of the study, Castagno and Khalifa's (2020) study with health professionals state that only 5% of the participants use AI applications. In our study, when the literacy levels of those who have heard and used AI applications are analyzed, the difference in both subdimensions and total scale score stands out. It is possible to say that hearing and using AI affect the literacy level. In studies conducted with various groups, it is reported that there is a relationship between hearing/using AI and knowledge level (Allam et al. 2023; Bock, Wolter, and Ferrell 2020; Park et al. 2019; Stewart et al. 2023; Wagner et al. 2021). These data suggest that encountering AI affects learning and use. In this context, in order for perioperative nurses to learn and use AI, which is gaining more and more place in the field of health every day, in‐service training, seminars, conferences, and so forth, training programs can be organized to increase awareness of AI in nurses, and their participation in existing programs can be encouraged.

Of the nurses participating in the study, 78.0% stated that AI would facilitate nursing practices. Similarly, in a study conducted with health professionals, 79% of the participants stated that AI would reduce the burden on health services (Castagno and Khalifa 2020). Similarly, the study data reports that health professionals believe that AI will reduce their workload, and this rate varies between 75% and 83%. (Castagno and Khalifa 2020; Codari et al. 2019; Oh et al. 2019). This view in the study data and the literature brings with it the view that nurses' knowledge and usage skills of AI should also increase. In this context, for AI to contribute to health services, knowledge, and skills in usage should be increased.

Existing limitations should be taken into consideration when interpreting the study data. Since the study was conducted only with perioperative nurses in Türkiye, it would not be appropriate to generalize to nurse populations working in different units and countries. In addition, snowball sampling method was used in the study in order to facilitate access to a hard‐to‐reach study group. Although this method is effective in the process of collecting data for a specific group, it also brings some limitations such as incompleteness and prejudice in representing the universe. The data obtained from the study are quantitative and do not provide qualitative information.

Existing limitations should be taken into consideration when interpreting the study data. Since the study was conducted only with perioperative nurses in Türkiye, it would not be appropriate to generalize to nurse populations working in different units and countries. In addition, snowball sampling method was used in the study in order to facilitate access to a hard‐to‐reach study group. Although this method is effective in the process of collecting data for a specific group, it also brings some limitations such as incompleteness and prejudice in representing the universe. The lack of multivariable analyses in the study creates a limitation in identifying underlying relationships or complex factors. This limitation should be considered when interpreting the results. The data obtained from the study are quantitative and do not provide qualitative information.

## 5 Conclusions and Recommendations

This study presents data on the AI literacy levels of perioperative nurses and related factors and reveals that the AI literacy level of perioperative nurses is at a medium level. Those who hear and use AI applications think AI will reduce the workload and that perioperative nurses who use technology tools have higher levels of AI literacy. In addition, as age increases, the level of AI literacy decreases. It should be ensured that disadvantaged groups in terms of the level of AI literacy in the study (women, older age, nonusers of AI, etc.) are supported. Younger nurses may exhibit higher AI literacy due to greater familiarity with technology, while those with more experience may require targeted training. Gender‐related differences could reveal varying comfort levels or perceptions of AI, which could guide the development of more tailored educational programs. We aim to address these subgroup comparisons in future research to refine our approach and better meet the diverse needs of perioperative nurses. The development of projects to increase nurses' knowledge and use of AI can be suggested, as can the inclusion of AI use in nursing education curricula and in the topics that should be covered in in‐service training. Data collection tools can be developed to obtain accurate data in measuring nurses' AI knowledge and use levels. These findings may also contribute to developing training programs and practice guides that will support nurses working in different health systems to use AI tools effectively. This can help to strengthen the professional skills of nurses in different systems. In particular, items such as the ethical dimensions of AI use and evaluation processes can be adapted under the international knowledge level of nurses and the technological infrastructure of local health systems. It is essential to safeguard patient data against unwanted access when AI‐based systems conduct analyses. For instance, if a postsurgical problem prediction system compromises patient confidentiality, nurses are obligated to identify and rectify the breach. Furthermore, when AI‐assisted systems render erroneous conclusions (e.g., inaccurately categorizing a patient's risk level), nurses may encounter ambiguity regarding the allocation of ethical responsibility. These circumstances affect both patient safety and professional responsibility.

## 6 Relevance for Clinical Practice

The study's findings indicate the necessity of developing specialized educational programs and in‐service training to improve the AI literacy of perioperative nurses. Training courses must encompass subjects including surgical risk evaluation, patient surveillance, and the ethical implications of AI in healthcare. Experiential learning techniques, such as laboratory experiments, simulations, and digital training, can enhance the acquisition of skills essential for incorporating AI into clinical practice. Furthermore, integrating AI content into nursing curricula and providing ongoing professional development opportunities will guarantee that nurses remain abreast of evolving technology. This strategy will enhance nurses' preparedness to utilize AI proficiently, elevate patient care, and streamline clinical procedures.

## Author Contributions

**Hilal Kahraman:** conceptualization, investigation, funding acquisition, writing – original draft, methodology, formal analysis, resources, project administration, writing – review and editing. **Seda Akutay:** writing – review and editing, methodology, validation, visualization, formal analysis. **Hatice Yüceler Kaçmaz:** methodology, conceptualization, supervision, formal analysis. **Sultan Taşci:** supervision, methodology, conceptualization.

## Ethics Statement

Before starting the research, Academic Board Permission from Erciyes University Faculty of Health Sciences and Erciyes University Social and Human Research Ethics Committee Permission (number: 2024/68, date: 27.02.2024) was obtained. The Informed Voluntary Consent Form (ICVF) prepared for the nurses included in the study was presented, and a box for participation was created. Access to the survey questions was provided if the nurses approved the consent box.

## Conflicts of Interest

The authors declare no conflicts of interest.

## Data Availability

The data that support the findings of this study are available from the corresponding author upon reasonable request.
